# Linc01105 acts as an oncogene in the development of neuroblastoma

**DOI:** 10.3892/or.2021.8156

**Published:** 2021-07-26

**Authors:** Mujie Ye, Jing Ma, Baihui Liu, Xiangqi Liu, Duan Ma, Kuiran Dong

Oncol Rep 42: 1527-1538, 2019; DOI: 10.3892/or.2019.7257

Subsequently to the publication of the above article, the authors have found that Fig. 4A on p. 1532 contained some errors. Owing to mistakes made during the preparation and revision of the manuscript, the invasion assay data images selected to show both the ‘Control’ and ‘shRNA2’ groups of the invasion and migration experiments were derived from the same original sources.

A corrected version of the Fig. 4, showing the correct data for the invasion and migration assay experiments with the Control and shRNA2 groups, is shown below. These inadvertent errors did not affect the conclusions reported in this paper, and all the authors agree with this Corrigendum. The authors thank the editor of *Oncology Reports* for presenting them with the opportunity to publish this Corrigendum, and apologize to the editor and to the readership of the journal for any inconvenience caused.

## Figures and Tables

**Figure 4.**
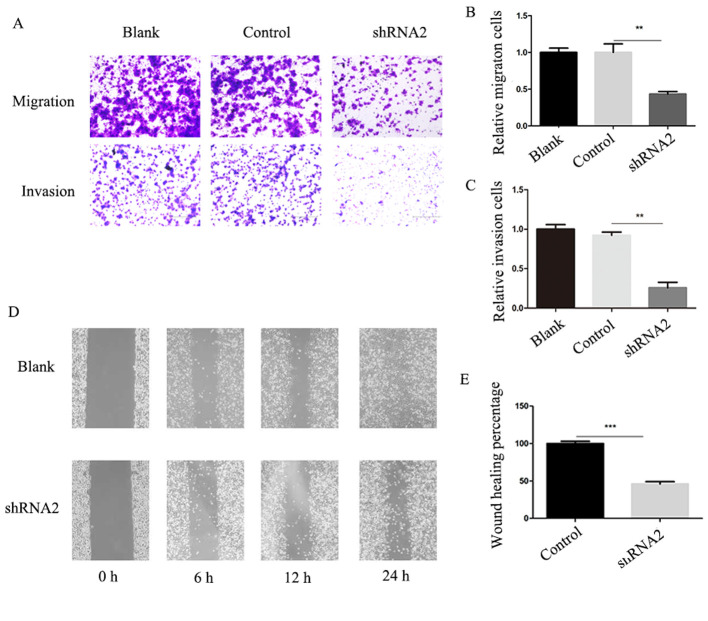
Silencing of linc01105 inhibits migration and invasion in SH-SY5Y cells. (A) Representative images of Transwell migration and invasion assays. (B) Quantification of migration. (C) Quantification of invasion. (D) Representative images and (E) quantification of wound closure assay. **P<0.01, ***P<0.005 compared with control. shRNA, short hairpin RNA.

